# Computational analysis of protein–protein interactions of cancer drivers in renal cell carcinoma

**DOI:** 10.1002/2211-5463.13732

**Published:** 2023-11-27

**Authors:** Jimin Pei, Jing Zhang, Qian Cong

**Affiliations:** ^1^ Eugene McDermott Center for Human Growth and Development University of Texas Southwestern Medical Center Dallas TX USA; ^2^ Department of Biophysics University of Texas Southwestern Medical Center Dallas TX USA; ^3^ Harold C. Simmons Comprehensive Cancer Center University of Texas Southwestern Medical Center Dallas TX USA

**Keywords:** cancer drivers, chromatin remodeling, protein–protein interaction, renal cell carcinoma, ubiquitination

## Abstract

Renal cell carcinoma (RCC) is the most common type of kidney cancer with rising cases in recent years. Extensive research has identified various cancer driver proteins associated with different subtypes of RCC. Most RCC drivers are encoded by tumor suppressor genes and exhibit enrichment in functional categories such as protein degradation, chromatin remodeling, and transcription. To further our understanding of RCC, we utilized powerful deep‐learning methods based on AlphaFold to predict protein–protein interactions (PPIs) involving RCC drivers. We predicted high‐confidence complexes formed by various RCC drivers, including TCEB1, KMT2C/D and KDM6A of the COMPASS‐related complexes, TSC1 of the MTOR pathway, and TRRAP. These predictions provide valuable structural insights into the interaction interfaces, some of which are promising targets for cancer drug design, such as the NRF2‐MAFK interface. Cancer somatic missense mutations from large datasets of genome sequencing of RCCs were mapped to the interfaces of predicted and experimental structures of PPIs involving RCC drivers, and their effects on the binding affinity were evaluated. We observed more than 100 cancer somatic mutations affecting the binding affinity of complexes formed by key RCC drivers such as VHL and TCEB1. These findings emphasize the importance of these mutations in RCC pathogenesis and potentially offer new avenues for targeted therapies.

## Graphical abstract

Renal cell carcinomas (RCCs) encompass a diverse group of kidney tumors. We employed AlphaFold to predict and model protein–protein interactions for RCC drivers and assessed the effects of cancer somatic mutations found in the interaction interfaces. AlphaFold models revealed high‐confidence interactions involving various RCC drivers. Novel interfaces such as NRF2‐MAF1 provide potential targets for development of cancer therapeutics.

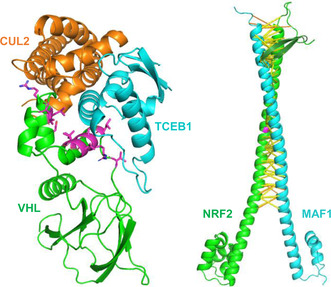

**Abbreviations**
ccRCC: clear cell renal cell carcinoma
chRCC: chromophobe RCC
COMPASS: COMplex of Proteins Associated with SET1
CUL3: Cullin 3
HIF‐α: α‐subunit of hypoxia‐inducible factor
HIF‐β: β‐subunit of hypoxia‐inducible factor
KEAP1: Kelch‐like ECH‐associated protein 1
KMT2: type 2 lysine methyltransferase
MTOR: mechanistic target of rapamycin
NCG: Network of Cancer Genes
NFE2L2: nuclear factor (erythroid‐derived 2)‐like 2
PAE: Predicted Aligned Error
PDB: Protein Data Bank
PIKK: phosphatidylinositol 3‐kinase‐related kinase
pLDDT: predicted Local Distance Difference Test
PMID: PubMed Identifier
PPI: protein–protein interaction
pRCC: papillary RCC
RCC: renal cell carcinoma
SAGA: Spt‐Ada‐Gcn5 acetyltransferase complex
SLiM: short linear motif
TKI: tyrosine kinase inhibitor
TRRAP: Transactivation/tRansformation‐domain Associated Protein
TSG: tumor suppressor gene

Kidney cancer ranks among the 10 most prevalent cancers in the United States [1] and caused about 180 000 deaths globally in 2020 [2]. Cases of the most common kidney cancer, renal cell carcinoma (RCC), have been on the rise in recent years [3, 4]. While early diagnosis and intervention by urological procedures could successfully treat RCCs, the overall prognosis remains poor, particularly for late‐stage patients. Detection of early‐stage RCCs is hindered by the lack of noticeable clinical symptoms in most cases. Furthermore, traditional drugs for RCCs, such as tyrosine kinase inhibitors (TKIs), have demonstrated limited efficacy. RCCs comprise a heterogeneous group of kidney tumors, with clear cell RCC (ccRCC) accounting for 70–80% of cases, papillary RCC accounting for 10–15% of cases, and chromophobe RCC accounting for 3–5% of cases [5]. Precision medicine has made significant progress in targeted therapy of RCCs by differentiating between these subtypes in genomic and pathway contexts [3, 6]. It is crucial to advance our understanding of the molecular mechanisms underlying RCC, as the knowledge could pave the way for innovations in diagnosis and treatment strategies.

The development and progress of cancer often result from the deregulation of multiple cellular processes that leads to unrestricted proliferation and replication potentials, insensitivity and resistance to antigrowth signals and apoptosis, promotion of angiogenesis, and the capability of invasion and metastasis [7]. Protein complexes formed by protein–protein interactions (PPIs) play critical roles in diverse aspects of cellular processes [8], including those related to cancer. Targeting cancer‐enabling PPIs has emerged as a promising strategy in drug development, aiming to disrupt these interactions and hinder tumor growth [9, 10, 11]. While large‐scale high‐throughput experimental studies have generated large datasets of human PPIs at the whole proteome level [12, 13, 14] or on cancer‐related proteins [15, 16], these datasets often suffer from high rates of false positives and false negatives with considerable inconsistencies among them [17, 18].

Cancer is often driven by genetic alterations that activate oncogenes or deactivate tumor suppressor genes [19]. These alterations can manifest as structural changes in chromosomes, gene fusion events, changes in gene copy numbers, and mutations at specific nucleotide positions. Next‐generation sequencing has been employed to identify genetic differences between cancer and normal cells [20]. Cancer genomic studies have expanded the landscape of cancer driver genes and their genetic alterations and advanced our understanding of the molecular mechanisms of cancer growth, metastasis, and drug resistance [21, 22, 23, 24, 25]. Detection of disease‐causing genetic variations or somatic mutations in cancer cells by genome sequencing provides valuable information for making clinical decisions for cancer patients [26]. A significant fraction of germline and somatic mutations occurs at the PPI interfaces [27, 28]. These mutations can have profound functional and clinical implications by disrupting protein complexes and interrupting essential cellular processes such as signal transduction and protein degradation [29, 30]. Accurate prediction of PPI interfaces holds tremendous potential in interpreting the clinical significance of cancer mutations and identifying targets for cancer drug development. By understanding how these mutations affect protein interactions, we can gain deeper insights into the underlying mechanisms of cancer and identify novel therapeutic avenues.

Deep‐learning methods have revolutionized our ability to predict high‐resolution protein structures [31] and model protein–protein interactions [32]. Methods such as AlphaFold [31] and RoseTTAFold [33] have been used to identify PPIs and model the structures of their interfaces with an accuracy better than large‐scale experimental studies [34]. These deep‐learning methods can be applied to screen confident PPIs from noisy high‐throughput candidate PPIs identified in large‐scale studies and obtain high‐quality structure models for the protein complexes [32, 34, 35].

In this study, we investigate PPIs involving RCC driver proteins and the effects of cancer somatic mutations at the PPI interfaces. We collected a comprehensive set of RCC drivers from publicly available databases [36, 37]. AF2Complex [38], FoldDock [39], and an in‐house program [40, 41, 42], all based on AlphaFold, were utilized to predict protein complexes formed by these cancer drivers. These methods predicted hundreds of PPIs formed by various RCC drivers and offered novel structural insights into protein complexes with important roles in RCC progression, including complexes involving the protein interaction hub proteinTCEB1, the KMT2C/D COMPASS complex, the TRRAP complexes, and the TSC complex of the MTOR pathway. We also identified RCC somatic mutations occurring at the interfaces of predicted and experimental structures of the protein complexes formed by RCC drivers. We evaluated their functional impact in terms of binding affinity changes. Cancer mutations affecting the binding affinity of protein complexes were mostly observed in several key RCC drivers, such as VHL and TCEB1, while few deleterious missense mutations were observed in other RCC drivers, such as those involved in chromatin remodeling.

## Results and Discussion

### Functional categories of RCC drivers

We collected a set of canonical and candidate RCC driver proteins from two resources: the Network of Cancer Genes (NCG) [36] and OncoVar [37] (see Materials and methods). Canonical cancer drivers mainly fall into several functional categories: protein degradation and ubiquitination, chromatin remodeling, transcription, MTOR pathway, signaling, cell cycle, and immunity (Table 1). The functional category of chromatin remodeling includes the largest number of NCG canonical RCC cancer drivers (bold names in Table 1). They include large, modular proteins with enzymatic domains that perform post‐translational modifications of histones such as SETD2, KDM5C, KDM6A, KMT2C, and KMT2D, as well as nonenzymatic subunits of large chromatin remodeling complexes such as PBRM1, ARID1A, ARID2, and SMARCB1 [43]. Several canonical RCC drivers (BAP1, CUL3, KEAP1, and VHL) are involved in ubiquitin‐related pathways such as protein degradation. In particular, *VHL* is the most frequently mutated gene in ccRCC (the dominating subtype of RCC) [44]. Four NCG canonical cancer drivers (FLCN, MTOR, TSC1, and TSC2) and two OncoVar cancer drivers (TMEM127 and RHEB) are involved in the MTOR pathway that regulates metabolism and is frequently perturbed in RCCs [45]. The functional categories of transcription and signaling are two other major contributors of NCG canonical cancer drivers of RCCs, while the functional categories of cell cycle and immunity are less represented in RCCs (Table 1). One characteristic of RCCs compared with other cancer types is that most canonical drivers of RCCs are encoded by tumor suppressor genes (TSGs, marked by (s) in Table 1). Only seven NCG canonical drivers are encoded by oncogenes (marked by (o) in Table 1), including three protein/lipid kinases—MTOR, MET, and PIK3CA.

**Table 1:** Functional categories of RCC drivers. These drivers are classified in the NCG and OncoVar databases. NCG canonical cancer drivers are in bold. OncoVar cancer drivers are underlined. NCG candidate cancer drivers are in regular font. Oncogenes and tumor suppressors classified in NCG or OncoVar are labeled by (o) and (s), respectively. These drivers are classified according to broad functional categories.

Functional category	RCC drivers
Protein ubiquitination and degradation	**VHL** (s), **BAP1** (s), **CUL3** (s), **KEAP1**(s), TCEB1/ELOC, CUL7, ASB15, WDFY3
Chromatin remodeling	**ARID1A** (s), **ARID2**(s), **KDM5C** (s), **KDM6A** (s), **KMT2C** (s), **KMT2D** (s), **PBRM1** (s), **SETD2** (s), **SMARCB1** (s), DNMT3A(o), CHD3
Transcription	**CRTC1**(o), **ERCC2**(s), **NF2** (s), **NFE2L2**(o), **TP53** (s), **TRRAP**(o), HIF1A(o), RB1(s), MAML2(o), MAX(s), NONO, SFPQ(s), SPEN(s), TFE3(o), TFEB(o), CNOT1, MED13, TRIM37, ZFHX4, ZNF469, ZNF765, ZNF804A
MTOR pathway	**FLCN** (s), **MTOR** (o), **TSC1** (s), **TSC2** (s), TMEM127(s), RHEB
Other signaling pathways	**FAT1**(s), **FAT3**, **LRP1B**(s), **MET** (o), **PIK3CA** (o), **PTEN** (s), PTK6(o), TRIO
Cell cycle	**ATM** (s), **STAG2** (s), CDKN1A(s), PRCC, CCNB2
Immunity	**CARD11** (o), BTNL3, NAV3
Others	CLTC(s), FH(s), HNRNPM, NEFH, AHNAK, AKAP13, AKAP9, ANPEP, C2CD4C, CCDC168, COL11A1, CPQ, CSMD3, CUBN, FAAH2, FAM111B, FMN2, GABRB2, LRRK2, MICALCL, MSR1, NPNT, PDHB, PDXDC1, PIEZO2, PLEC, PNKD, RADIL, RYR1, SHANK1, SLC1A3, SLC9A3R1, SLITRK6, SPTBN4, SYNE2, TXNIP

### Comparison of AlphaFold‐based methods of PPI predictions for RCC drivers

We applied three protein complex prediction methods (AF2Complex [38], FoldDock [39], and an in‐house program based on the contact probability of AlphaFold, namely AF‐contact [40, 41, 42]) to predict the protein–protein interactions involving RCC drivers. All these methods were developed based on AlphaFold, and they mainly differ in the evaluation of complex structures. Our method, AF‐contact, relies on the inter‐residue distograms (probability distribution over a series of distance bins) produced by AlphaFold, and the sum of probability in bins corresponding to < 12 Å is used as the inter‐residue contact probability. AF‐contact utilizes the intermolecular contact probability for ranking the protein complex models (see Materials and methods). AF2Complex relies on an interface score that combines PAE (Predicted Aligned Error) estimates of interface residues and the size of the interface. FoldDock has a scoring metric that combines the interface pLDDT (predicted Local Distance Difference Test) score and the number of interface contacts.

**Fig. 1:**
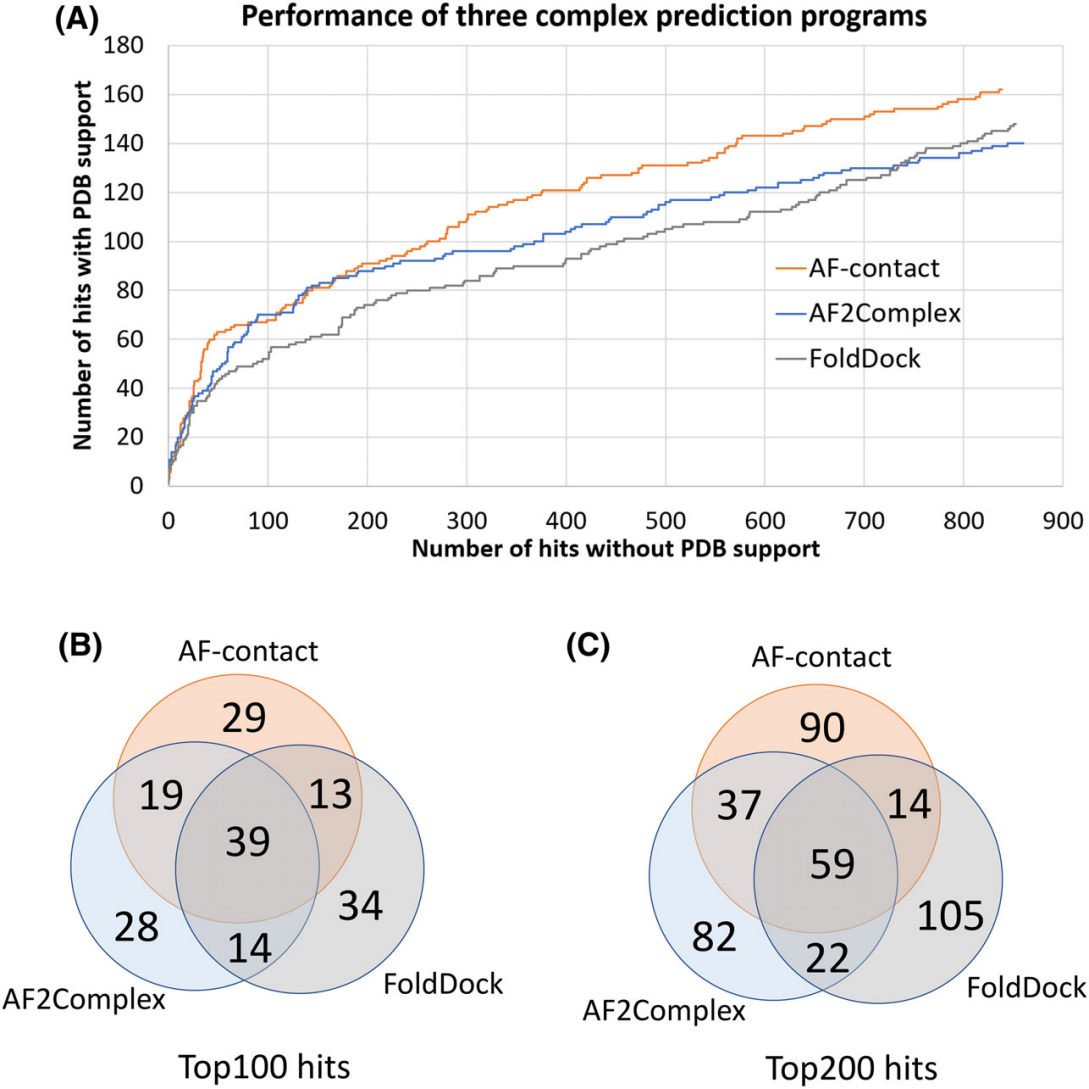
Comparison of protein complex prediction by three programs (AF‐contact, AF2Complex, and FoldDock). (A) The numbers of predicted PPIs supported by experimental structures in PDB among the top 1000 predictions of the three programs. (B) Venn diagram of the top 100 predicted PPIs by the three programs. (C) Venn diagram of the top 200 predicted PPIs by the three programs.

We applied these methods to the 3595 candidate PPIs involving RCC drivers (see below), and we compared their performance by examining the fraction of pairs with experimentally determined structures in PDB among the top‐ranking predictions. While the set of candidate PPIs may still contain a considerable fraction of false PPIs, those supported by experimental structures are highly confident and can be used as positive controls. We ranked the predicted PPIs by each method's PPI confidence metric (contact probability for AF‐contact, interface score for AF2Complex, and pDockQ for FoldDock), and the numbers of predicted PPIs by each method that are supported by PDB structures at each rank, up to top 1000, are shown in Fig. 1A. These curves show that the three programs perform similarly for top‐scoring pairs. Among the top 50 predicted PPIs, AF‐contact, AF2Complex, and FoldDock have 30, 30, and 29 pairs supported by experimental structures, respectively. Among the top 100 predictions, AF‐contact has 60 pairs supported by PDB structures, while AF2Complex and FoldDock have 49 and 48 such pairs, respectively. The number of pairs with experimental structures grows much slower after the top 100 hits (Fig. 1A). AF‐contact performs slightly better as the number of PDB‐supported predictions is mostly higher than the other two methods at different ranks.

We also compared the top‐scoring PPIs predicted by the three programs, revealing a high consistency between them. For example, 39 PPIs (~ 40%) were predicted by all three programs among their top 100 predictions (Fig. 1B). Approximately one‐third of the top 100 predicted PPIs generated by each program was unique and did not overlap with the top 100 predictions of the other two programs. The consistency between the programs decreased when more predictions were included in the comparison (Fig. 1C). Among the top 200 predictions, only 59 (~ 30%) were consistently found by all three pairs. AF‐contact and AF2Complex appear to show more consistent results with each other and share more top predictions.

### Prediction of PPIs for RCC drivers and assessment of mutation effects

Candidate human PPIs involving RCC drivers were obtained from the BioGRID database [46]. Since most of these PPIs were identified from large‐scale experiments prone to false positives [17, 18], we selected 3595 candidate PPI pairs involving RCC drivers based on publication‐weighted scores (see Materials and methods). Our method AF‐contact predicted high‐confidence models for 211 pairs of proteins (299 domain pairs with contact probability > 0.9). Among those, 85 protein pairs are supported by experimental structures. The majority of these predictions are supported with high confidence by the other two programs: AF2Complex and FoldDock (Table S1). Most of the examples discussed below are supported by two or more programs.

We mapped the RCC somatic mutations to predicted and experimentally determined PPI interfaces involving RCC drivers. These mutations are from the COSMIC database [23] and a large collection of somatic mutations from 148 RCC patients [47]. The effects of mutations found at PPI interfaces were accessed by BindProfX with the FoldX as the force field [48, 49]. Predicted free energy change associated with each mutation at the PPI interface is provided in Table S2, and mutations predicted to destabilize protein complexes remarkably (ΔΔG > 1.4 kcal·mol^−1^) are highlighted in yellow. These predictions were used to interpret the functional impact of cancer somatic mutations discussed below.

### VHL mutations affect binding affinities in multiple protein complexes

Biallelic inactivation of the tumor suppressor gene *VHL* is a hallmark in most ccRCCs [50]. *VHL* inactivation is often due to the loss of chromosome 3p and point mutations. VHL is the substrate‐binding subunit of an E3 ubiquitin ligase that recognizes and targets the α‐subunit of hypoxia‐inducible factor (HIF‐α) for protein degradation in physiological conditions with normal oxygen level [51]. In hypoxic conditions, HIF‐α is not hydroxylated or targeted by VHL for degradation. The stable HIF‐α forms a dimer with the β‐subunit (HIF‐β, also called ARNT) that functions as a transcription factor to promote the expression of target genes involving many cellular processes in adaptation to hypoxic conditions such as glycolysis, glucose transport, and angiogenesis. These functions support the critical role of VHL in regulating tumor progression in ccRCC.

**Fig. 2:**
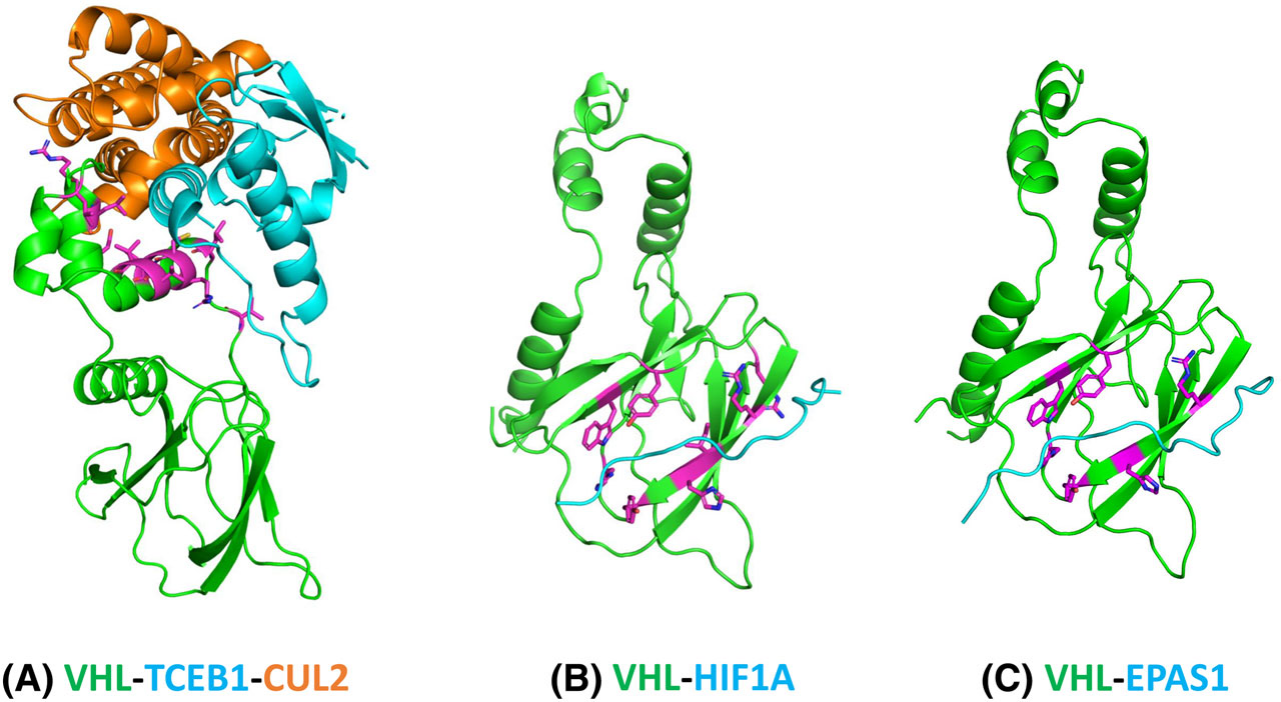
Protein–protein interactions of VHL. (A) The VHL‐TCEB1‐CUL2 complex (based on PDB id: 4wqo). (B) The VHL‐HIF1A complex (based on PDB id: 1lm8). (C) The VHL‐EPAS1 complex (based on PDB id: 6i7q). Predicted destabilizing interface mutations (ΔΔG > 1.4 kcal·mol^−1^) on VHL were shown in magenta with the sidechains displayed in sticks.

VHL forms protein complexes with TCEB1 (ELOC), ELOB, and CUL2. While the VHL‐TCEB1 interaction was detected by AlphaFold, the VHL‐CUL2 interaction was not detected. We mapped several deleterious mutations of VHL to the interface of the experimentally determined structure of the VHL‐TCEB1‐CUL2 complex [52] (PDB id: 4wqo, Fig. 2A). These mutations are located in the small helical SOCS‐box domain of VHL.

Experimental structures have been determined for the complexes of VHL‐HIF1α and VHL‐EPAS1 (HIF2α) [53, 54]. Both HIF1α and EPAS1 utilize a short and extended segment with a hydroxylated proline to interact with the immunoglobulin domain of VHL at similar locations (Fig. 2B,C). AlphaFold did not detect the interactions of VHL‐HIF1α and VHL‐EPAS1 above the probability score cutoff (0.9), possibly due to the small interfaces and the inability of AlphaFold to incorporate modified residues (hydroxylated prolines) in the modeling process. Several deleterious VHL mutations were mapped in the VHL‐HIF1α interface (PDB id: 1lm8) and the VHL‐EPAS1 interface (PDB id: 6i7q; Fig. 2B,C) [53, 54].

### TCEB1 mutations in multiple PPI interfaces

**Fig. 3:**
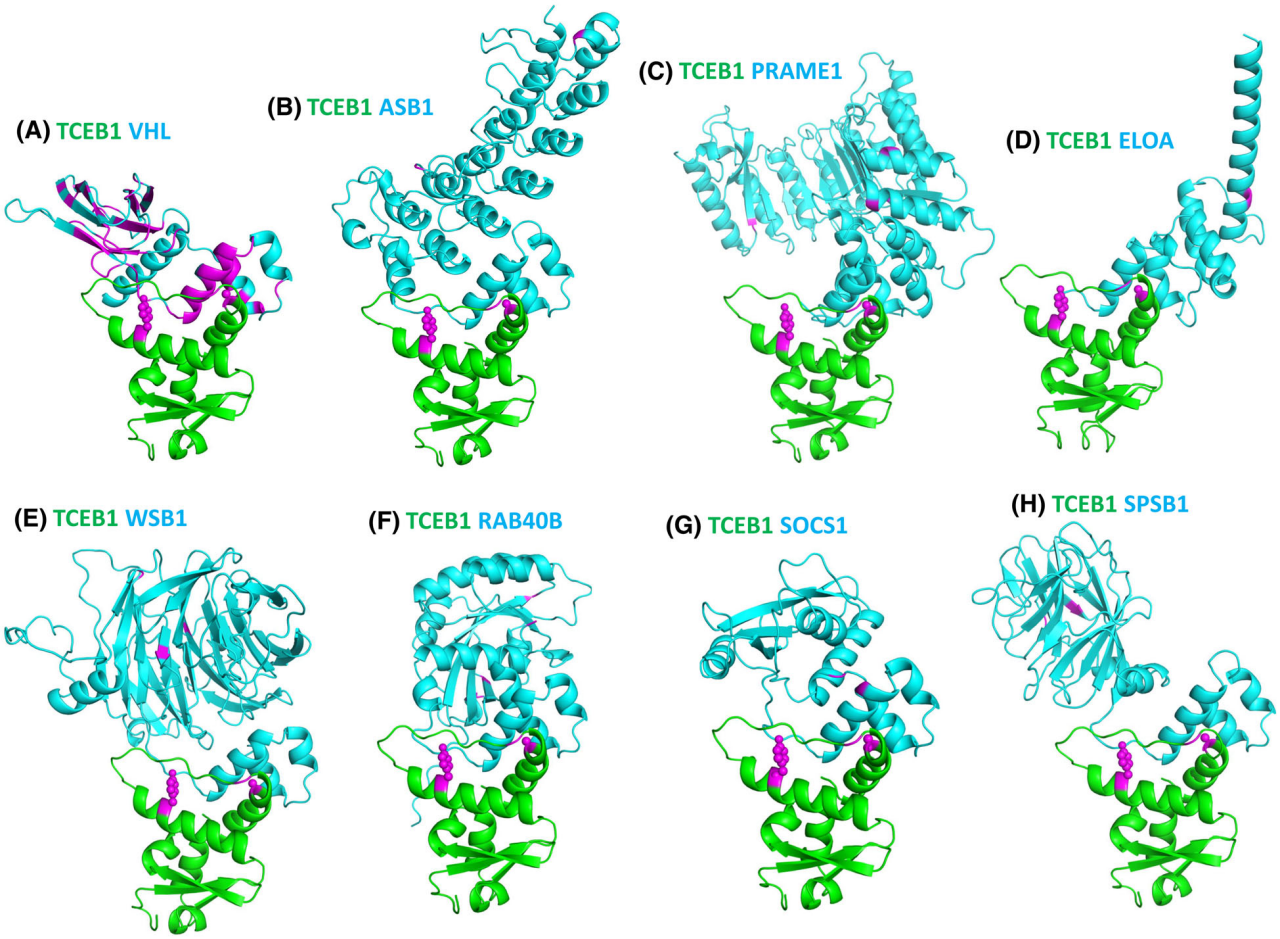
Protein–protein interactions of TCEB1 predicted by AlphaFold. (A) The TCEB1‐VHL complex. (B) The TCEB1‐ASB1 complex. (C) The TCEB1‐PRAME1 complex. (D) The TCEB1‐ELOA complex. (E) The TCEB1‐WSB1 complex. (F) The TCEB1‐RAB40B complex. (G) The TCEB1‐SOCS1 complex. (H) The TCEB1‐SPSB1 complex. RCC mutations in these proteins were shown in magenta. Sidechains of predicted destabilizing interface mutations (ΔΔG > 1.4 kcal·mol^−1^) on TCEB1 were shown in spheres.

TCEB1 (ELOC, elongin C) is a critical component of the VHL complex that targets HIF‐α for degradation. Recently, a distinct group of kidney cancers characterized by the loss of heterozygosity at the *TCEB1* location in chromosome 8 and hotspot mutations in *TCEB1* were identified by integrated sequence analysis [55]. These tumors exhibited different morphological and immunohistochemical characteristics compared with ccRCC tumors caused by *VHL* alterations. TCEB1 is a SOCS‐box binding protein [56]. Indeed, AlphaFold predicted the interactions between TCEB1 and numerous SOCS‐box‐containing proteins, such as VHL, SOCS1, ELOA, ASB1, PRAME1, and WSB1 (8 proteins shown in Fig. 3). The SOCS box is a short helical domain found in various proteins with different domain contents. Interaction through the SOCS box facilitates targeting these proteins by the adapter TCEB1 to E3 ubiquitin ligases for degradation [56]. While the VHL‐TCEB1 interaction has been well studied, the roles of TCEB1 interactions with other SOCS‐box‐containing proteins have not been fully explored. Deleterious RCC mutations in TCEB1 such as Y79C/S/F/N and A100P are mapped to interfaces of these complexes (Fig. 3), suggesting that these mutations could interfere with the degradation of various TCEB1 targets besides VHL. The specific roles of various TCEB1‐interacting SOCS‐box binding proteins in RCC progression remain to be studied.

### Structural insight into the COMPASS‐related complexes

RCC drivers KMT2C and KMT2D belong to the family of type 2 lysine methyltransferases (KMT2) involved in histone modification and chromatin remodeling [57]. They perform post‐translational methylation of histone 3 lysine 4 (H3K4), which is frequently associated with active transcription. KMT2C (4911 amino acid residues) and KMT2D (5537 amino acid residues) are large, modular proteins with methyltransferase activity driven by a conserved SET domain and contain a large fraction of disordered regions. They are part of the COMPASS (COMplex of Proteins Associated with SET1)‐related complexes conserved from fungi to metazoans [58, 59]. The other subunits of the COMPASS complexes include NCOA6 (also named ASC2), KDM6A (also named UTX), PAXIP1 (also named PTIP), PAGR1, NCOA1, WDR5, RBBP5, ASHL2, and DPY‐30. KDM6A, a demethylase, is another canonical cancer driver of ccRCC [60].

**Fig. 4:**
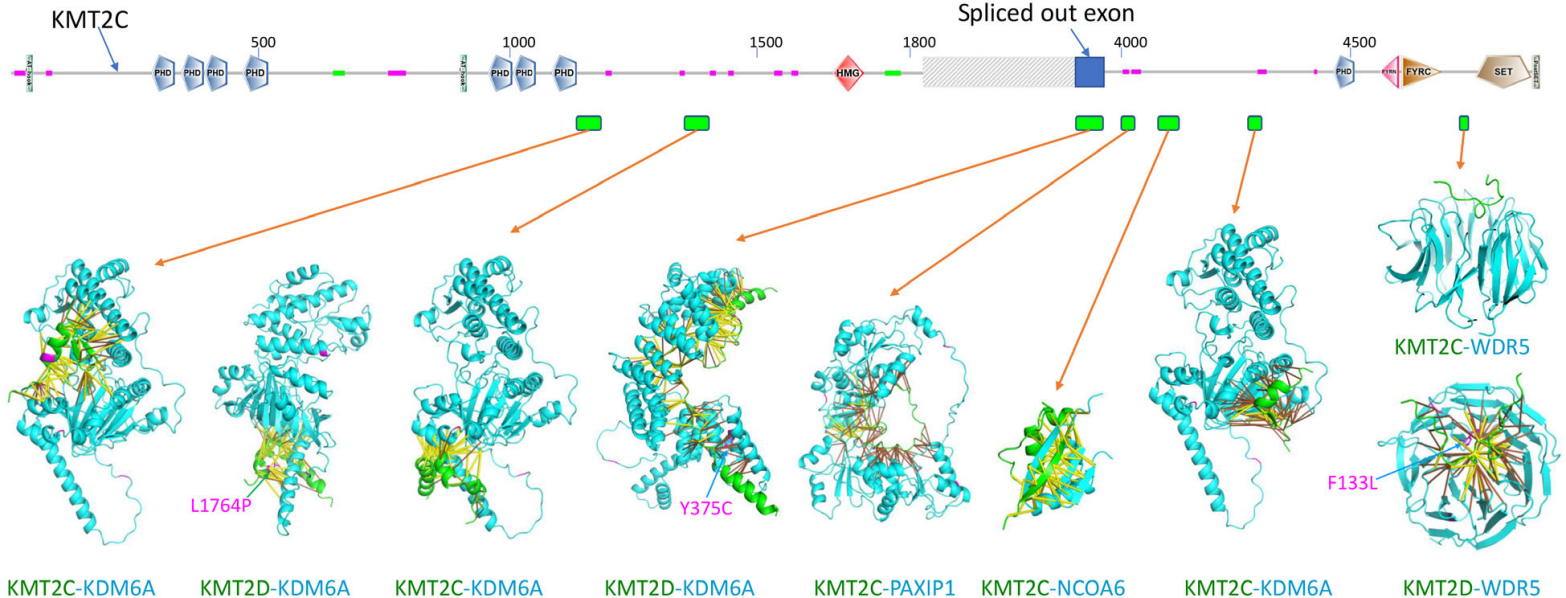
Protein–protein interactions of KMT2C/D in the COMPASS‐related complex. The domain diagram of KMT2C is shown with globular domains labeled according to the SMART database. The long and disordered region is omitted and replaced by a crossbar. The extra exon spliced out in the canonical form of KMT2C is shown as a blue rectangular box. The interaction regions are labeled by green boxes. KMT2C/D regions in the structural models were shown in green. The interaction partners of KMT2C/D are shown in cyan. Positions containing RCC missense mutations are shown in magenta. Contacts with scores above 0.9 are shown in yellow sticks, and contacts with scores between 0.5 and 0.9 are shown in orange sticks.

AlphaFold detected multiple interactions involving short linear motifs (SLiMs) in disordered regions of KMT2C/KMT2D and several other COMPASS‐related complex subunits such as KDM6A, NCOA6, and PAXIP1 (Fig. 4). Three SLiMs in KMT2C (residues 1167–1196, 1349–1388, and 4275–4298) interact with the demethylase domain of KDM6A (Fig. 4), suggesting that KMT2C could regulate the enzymatic activity of KDM6A. A SLiM in KMT2C (residues 4073–4120) forms a tightly packed small globular domain with residues 88–139 of NCOA6. Another SLiM in KMT2C (residues 3995–4040) interacts with four C‐terminal BRCT domains of the COMPASS complex subunit PAXIP1 (residues 589–1069). In addition, KMT2C uses a C‐terminal SLiM (residues 4703–4716) to interact with the WD40 domain of WDR5, where the complex structure has been solved [61].

A region (residues 4543–4618) in KMT2D, a paralog of KMT2C, is predicted to interact with the N‐terminal helical repeat domain of KDM6A (residue 38–405). Interestingly, the corresponding homologous region in KMT2C is encoded by an exon (shown in the KMT2C domain diagram in Fig. 4) that is spliced out in the canonical isoform of KMT2C (UniProt accession: Q8NEZ4). The protein segment encoded by this exon is present in two other KMT2C isoforms (UniProt: Q8NEZ4–2 and Q8NEZ4–3, 58 residues inserted between residues 3890 and 3891 of the canonical isoform). This suggests that the activity of the COMPASS complex could be modulated by alternative splicing.

Few RCC mutations were mapped to the interaction interfaces involving KMT2C (4 mutations) and KMT2D (6 mutations) despite their large sizes (Table S2). Three such mutations in the interaction interfaces of KMT2D (KMT2D L1764P, KDM6A Y375C, and WDR5 F133L) were predicted to be destabilizing (ΔΔG > 1.4 kcal·mol^−1^) according to free energy change calculations (labeled in Fig. 4). Due to the extensive interfaces involving the large subunits in the COMPASS‐related complexes, single amino acid mutations may not have a significant impact in disrupting their interactions. Inactivating mutations (nonsense, frameshift, or splice site disruption) in the subunits of COMPASS‐related complexes such as KMT2C, KMT2D, and KDM6A [62, 63] could in general have more dominant effects in the progress of RCCs than missense mutations.

### Protein complexes involving NRF2 (NFE2L2) and KEAP1

**Fig. 5:**
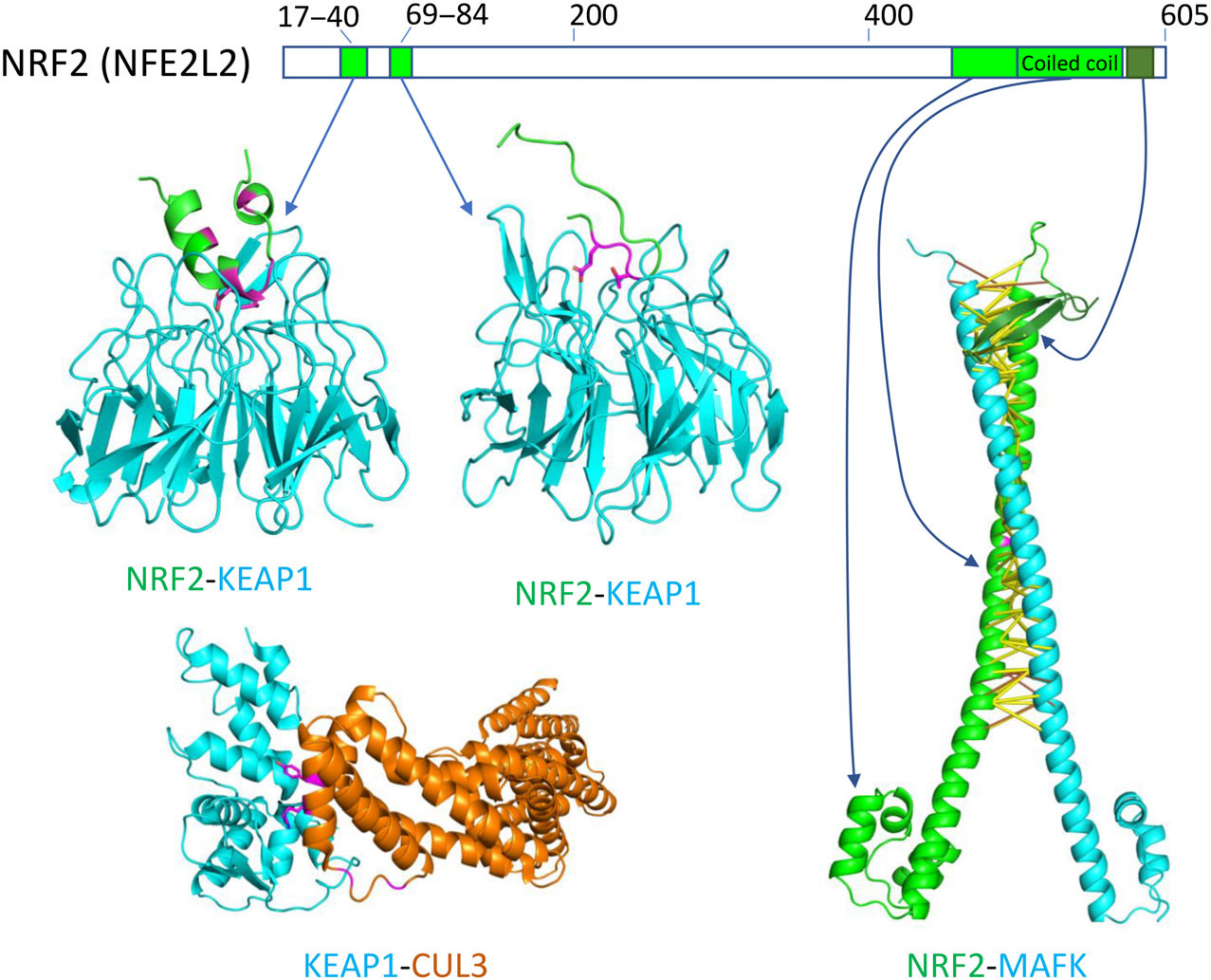
Protein–protein interactions of NRF2, KEAP1, and CUL3. NRF2 regions involved in PPIs are shown in green or dark green (C‐terminal β‐hairpin). The NRF2‐KEAP1 interactions are shown based on two experimental structures [PDB ids: 3wn7 (upper left) and 2flu (middle)]. The KEAP1‐CUL3 interactions are shown based on an experimental structure [PDB id: 5nlb (lower left)]. RCC mutations in these proteins are shown in magenta. Sidechains of predicted destabilizing interface mutations (ΔΔG > 1.4 kcal·mol^−1^) are shown in sticks. Contacts with scores above 0.9 are shown in yellow sticks, and contacts with scores between 0.5 and 0.9 are shown in orange sticks.

NRF2 (also named NFE2L2 for nuclear factor (erythroid‐derived 2)‐like 2) is a transcription factor that regulates the expression of multiple genes involved in cellular stress response [64]. NRF2 is targeted by protein degradation through its interaction with the Kelch‐like ECH‐associated protein 1 (KEAP1) [65], which is an adaptor protein that bridges NRF2 and Cullin 3 (CUL3)‐based E3‐ubiquitin ligase complex. Disruption of the NRF2‐KEAP1 interaction prevents NRF2 from degradation, and upregulation of NRF2 is a major driving event in certain RCCs and other types of cancers [66, 67]. NRF2, KEAP1, and CUL3 are classified as canonical RCC drivers in the NCG database. NRF2 utilizes two small SLiMs in disordered regions (residues 17–40 and residues 69–84) to interact with the beta‐propeller domain of KEAP1 [68, 69]. Several mutations in these regions [70], such as those involving Asp29, Leu30, and Gly31, have been found in RCCs. These mutations were predicted to destabilize the NRF2‐KEAP1 complex according to the calculation of free energy change (ΔΔG > 1.4 kcal·mol^−1^; Fig. 5). We also identified two predicted destabilizing RCC mutations in CUL3 (R59S and Y62H) in the experimentally determined interface of KEAP1 and CUL3 (Fig. 5) [71].

The transcriptional activity of NRF2 depends on the formation of the NRF2‐MAFK heterodimer [72]. AlphaFold predicted the interaction between NRF2 and MAFK with high contact probability (> 0.99). The complex model of NRF2 and MAFK demonstrates the interaction between the coiled coils from the two proteins, which forms a leucine zipper heterodimer. Moreover, the C terminus of NRF2 forms a β‐hairpin that also interacts with the coiled‐coil of MAFK (Fig. 5). No cancer somatic mutations with deleterious effects were identified in the NRF2‐MAFK interface in our datasets, reinforcing the idea that the NRF2‐MAFK interaction is critical for activating gene expressions for cancer progression. The newly discovered interface involving the NRF2 C‐terminal β‐hairpin presents a potential target for developing anticancer drugs. By disrupting the NRF2‐MAFK interaction, the transcriptional activity of NRF2 could be attenuated, offering a promising avenue for RCC drug design.

### Protein complexes in the mTOR pathway

The mTOR pathway is often perturbed in chromophobe RCC [73]. MTOR (mechanistic target of rapamycin), an evolutionarily conserved serine/threonine kinase, forms complexes such as mTORC1 and mTORC2 with other proteins to regulate cell growth and survival in response to nutritional status and various growth factors and stress signals [74]. MTOR phosphorylates downstream targets such as S6K1 to promote cell growth. Therefore, MTOR and its activator RHEB have been classified as oncogenes, while several negative regulators of the mTORC1 complex, such as TSC1, TSC2, TMEM127, and FCLN, are considered tumor suppressors [75, 76, 77].

**Fig. 6:**
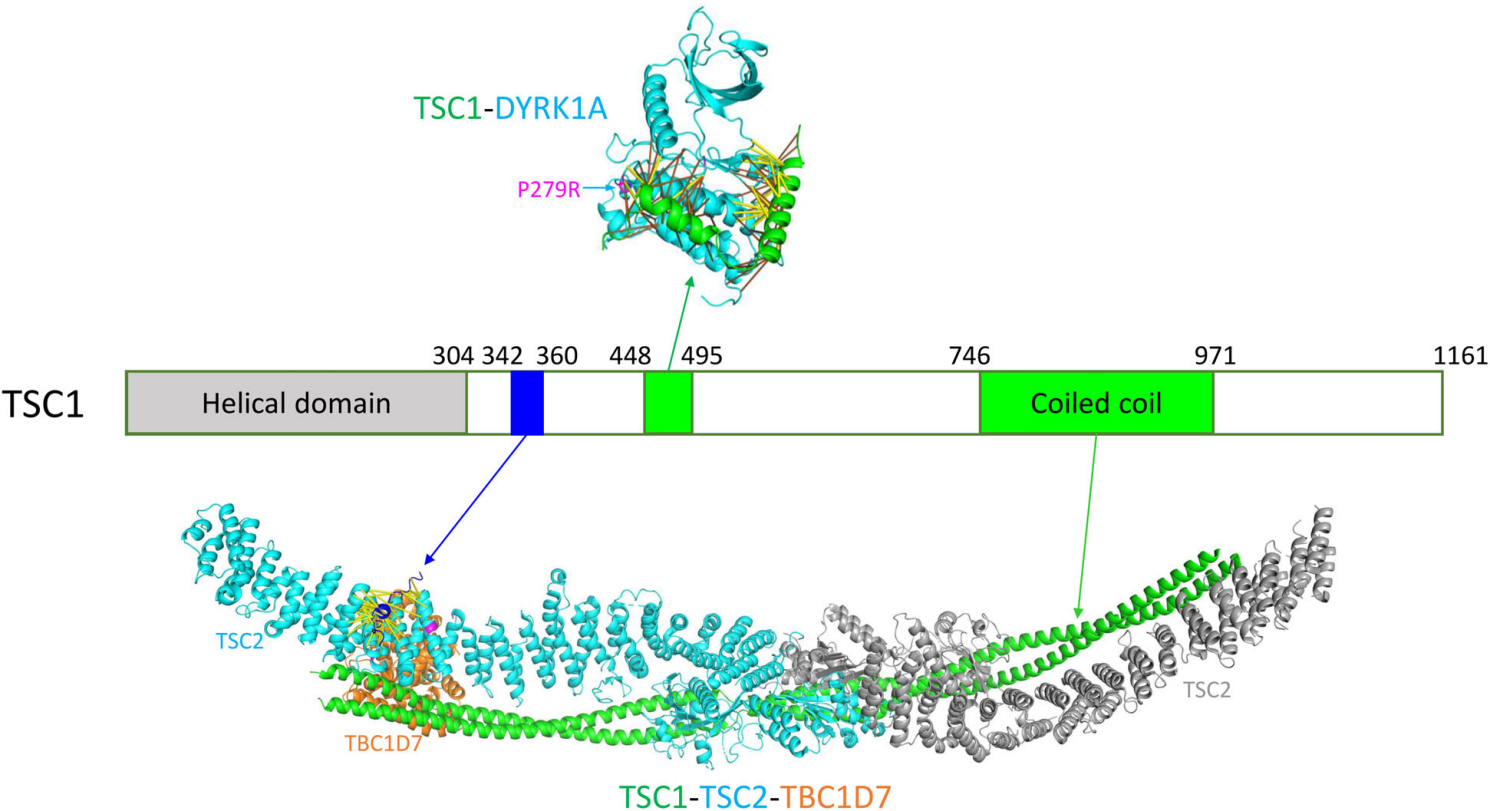
Protein–protein interactions of TSC1 in the mTOR pathway. The domain structure of TSC1 is shown with the N‐terminal helical domain and the coiled‐coil region labeled. The bottom structure is based on an experimental structural complex between TSC1, TSC2, and TBC1D7 (PDB id: 7dl2). Two copies of the coiled‐coil region of TSC1 form a dimer and are shown in green. The two subunits of the TSC2 dimer are shown in cyan and gray. A conserved region from 342 to 360 in TSC1 (shown in blue) was predicted to interact with TSC2, and the contacts between them with scores above 0.9 are shown in yellow sticks. The top structure shows the AlphaFold model of the complex between TSC1 (green) and DYRK1A (cyan). Contacts with scores above 0.9 are shown in yellow sticks, and contacts with scores between 0.5 and 0.9 are shown in orange sticks.

AlphaFold predicted several complexes involving proteins in the MTOR pathway, such as MTOR‐RPTOR, MTOR‐MLST8, and MTOR‐DEPTOR, most of which are supported by experimental structures [78]. The TSC complex, comprising TSC1, TSC2, and TBC1D7, is a major suppressor of MTOR. TSC1 possesses an N‐terminal helical repeat domain, disordered regions in the middle, and a C‐terminal coiled‐coil domain (Fig. 6). A recent structure of TSC1 in complex with TSC2 and TBC1D7 showed that the coiled‐coil region of TSC1 forms a homodimer that interacts with the elongated homodimer of TSC2 (Fig. 6). Part of the coiled‐coil region (residues 936–960) of TSC1 also interacts with TBC1D7 (Fig. 6). Interestingly, AlphaFold predicted an additional interaction site between TSC1 and TSC2 with high contact probability (> 0.99): A short sequence motif from TSC1 (residues 342–360, Fig. 6) embedded in the disordered region interacts with TSC2's HEAT repeat domain. This short sequence motif contains several highly conserved positions, such as W347, P349, and C353, that are mapped to the interaction interface, suggesting that the interaction through this motif could be functionally important for the TSC1‐TSC2 complex.

AlphaFold predicted the interaction between TSC1 and the protein kinase DYRK1A. This interaction has been reported in three high‐throughput experimental studies in BioGRID [46]. Moreover, it has been shown that DYRK1A regulates the mTOR pathway through binding to the subunits of the TSC complex [79]. However, the structural details of this interaction have not been revealed experimentally. AlphaFold model showed that the DYRK1A‐interacting segment in TSC1 lies in the disordered region. It forms two alpha helices surrounding the helical subdomain (C‐lobe) of the DYRK1A kinase domain (Fig. 6). The functional impact of this interaction has yet to be determined. One RCC somatic mutation in DYRKA1 (P279R) is mapped to the interaction interface and was predicted to destabilize (ΔΔG > 1.4 kcal·mol^−1^) this complex based on predicted free energy changes upon binding (Fig. 6).

### Structural insight into TRRAP complexes

Transactivation/tRansformation‐domain Associated Protein (TRRAP) is a PIKK (phosphatidylinositol 3‐kinase‐related kinase) family pseudokinase involved in signal transduction and transcriptional regulation [80]. TRRAP is a canonical cancer driver suggested by NCG and has been classified as an oncogene gene product in the COSMIC database [81]. Two other members in the PIKK family are also RCC drivers: ATM (a tumor suppressor) and MTOR (an oncogene product). TRRAP is a main component of two large macromolecular histone‐acetylation complexes: the Spt‐Ada‐Gcn5 acetyltransferase complex (SAGA) [82] and the nucleosome acetyltransferase of H4 complex (NuA4 or TIP60) [83].

**Fig. 7:**
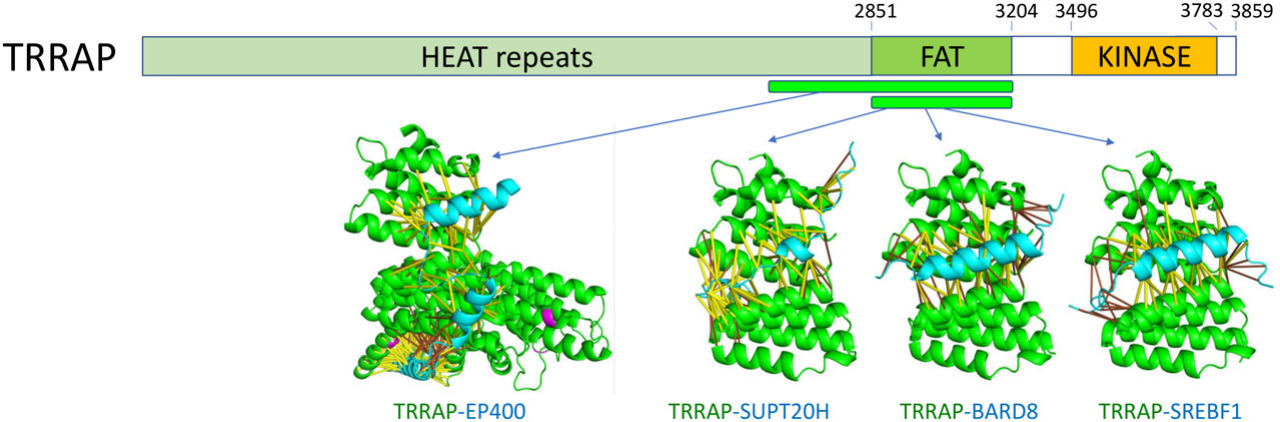
Protein–protein interactions of TRRAP. The domain structure of TRRAP is shown with globular domains labeled. TRRAP is shown in green in the protein complex structures. RCC mutations are shown in magenta. Contacts with scores above 0.9 are shown in yellow sticks, and contacts with scores between 0.5 and 0.9 are shown in orange sticks.

The NuA4 complex plays critical roles in transcriptional activation, primarily through acetylating histones H4 and H2A. Another key component of the NuA4 complex is EP400. AlphaFold predicted the interaction between TRRAP and EP400 with a high probability (score > 0.99). The interaction regions in TRRAP are from the HEAT domain of helical repeats and the FAT domain (Fig. 7). The region of interactions from EP400 does not form a globular domain. Instead, it contains several alpha helices that wrap around TRRAP helical repeats (Fig. 7).

AlphaFold also predicted the interaction between TRRAP and the SAGA complex core subunit SUPT20H—an α‐helix in SUPT20H docks onto the FAT domain of TRRAP (Fig. 7). The exact location on the TRRAP FAT domain is used in the interactions with two other proteins (BARD8 and SREBF1) and is also part of the interface between TRRAP and EP400 (Fig. 7). No somatic cancer mutations were identified in these TRRAP interfaces in our datasets. Since TRRAP has been classified as an oncogene product and could play a vital role in the transcriptional activation of genes promoting cell growth and cancer development, disrupting these interfaces could serve as a promising direction for cancer therapeutics targeting the TRRAP complexes.

## Conclusion

RCCs encompass a diverse group of commonly occurring kidney tumors. Numerous cancer driver proteins associated with different RCC subtypes have been identified, and the majority of them are encoded by tumor suppressor genes. RCC drivers can be categorized into major functional groups, including chromatin remodeling, protein degradation, and transcription regulation. To gain deeper insights into the function of these drivers, we employed AlphaFold to predict and model their interactions with other proteins. AlphaFold predictions revealed high‐confidence interactions involving various RCC drivers, including TCEB1, NRF2, subunits of the COMPASS‐related complexes, the TSC complexes in the MTOR pathway, and complexes involving TRRAP. Notably, many of these predicted complexes have not been structurally characterized. Therefore, these findings offer novel and valuable structural insights into the mechanisms underlying the functions of these complexes and their role in RCC development. A recurring scheme among these interactions is the utilization of short, linear motifs in disordered regions to interact with globular domains in other proteins. Cancer somatic missense mutations from large datasets of RCC genome sequencing were mapped to the interfaces of predicted and experimentally determined structures of the complexes. Cancer somatic mutations significantly impacted the stability of complexes formed by key RCC drivers, including VHL, TCEB1, and NRF2, highlighting the critical role of these mutations in RCC development. In contrast, relatively fewer deleterious missense mutations were found in other RCC drivers, especially those involved in chromatin remodeling. Furthermore, our analysis revealed a scarcity of cancer somatic mutations in the interfaces of complexes involving two oncogene products, NRF2 (the NRF2‐MAF1 interface) and TRRAP (interfaces involving the FAT domain of TRRAP), suggesting critical functions of these protein complexes in cancer cells. This observation holds promise for developing cancer therapeutics targeting the disruption of these interfaces.

## Materials and methods

### Collection of RCC drivers and identification of candidate RCC PPIs from databases

A set of RCC driver proteins were obtained from NCG [36] and OncoVar [37]. NCG has a manually curated set of cancer driver genes for various cancer types. Canonical drivers and candidate drivers classified by NCG were retrieved from these cancer subtypes: chromophobe RCC (chRCC), ccRCC, non‐clear cell RCC, papillary RCC (pRCC), and renal cancer (all histology). Cancer drivers were also retrieved from the OncoVar database for these RCC subtypes: ccRCC (KIRC), pRCC (KIRP), and chRCC (KICH). The combined set has 99 cancer drivers mapped to human‐curated entries in the UniProt database [84]. We obtained human PPIs involving these cancer drivers from the BioGRID database [46], which links each PPI to PubMed Identifiers (PMIDs), representing experimental studies supporting this interaction. We assigned a weight for each publication based on its associated number of interactions. The weight is defined as the reciprocal of the square root of the associated interaction number. The score for each interaction is defined as the sum of the weights of its associated PMIDs. We selected PPIs involving RCC drivers with scores no < 0.3 as candidate PPIs.

### Generation of protein sequence alignments

We used a sequence database of 49 102 568 proteins from 2568 representative or reference eukaryotic proteomes (one per species) downloaded from NCBI. For each human protein (referred to as the target protein below), the corresponding orthologous group at the eukaryotes level in OrthoDB [85] was identified. Human proteins that were not classified in OrthoDB or belonged to the Zinc finger C2H2‐type group (OrthoDB group: 1318335at2759), the Reverse transcriptase, RNaseH‐like domain group (OrthoDB group: 583605at2759), and the pentatricopeptide repeat group (OrthoDB group: 1344243at2759) were ignored as these groups contain a large number (over 58,000) of paralogous sequences from gene duplications. The set of OrthoDB orthologs for any target protein was clustered by CD‐HIT [86] at a 40% identity level. For each CD‐HIT cluster, we selected one representative sequence that showed the lowest BLAST [87] e‐value to the target protein. The representative sequences were then used as queries to search against the eukaryotic proteomes to identify homologous proteins to the target protein (e‐value cutoff: 0.001).

For each target protein, we identified a single best hit from each organism, if available, that satisfies two criteria: (a) its sequence identity to the target protein is above 35%; (b) it shows the highest sequence similarity to the target protein among the combined BLAST hits found by multiple representative sequences. These best hits and the target protein were aligned by MAFFT [88] (with the – auto option). We labeled each protein by the organism it belongs to in the resulting MSAs and removed positions that were gaps in the human proteins. We concatenated the aligned sequences of the same organism from two MSAs to construct the joint MSA for any two human proteins. In cases where a sequence is missing from an organism, we used gaps to replace that sequence and added these cases at the end of the joint MSA.

### Modeling protein complex structures with AlphaFold

We parsed large proteins into domains based on DeepMind's models of monomeric human proteins from the AlphaFold Protein Structure Database (https://alphafold.ebi.ac.uk/) as described previously [42]. AlphaFold was used to model protein complexes by inputting joint MSAs of two protein (or domain) alignments with a gap of 200 residues in between the alignments. Protein pairs with combined length ≤ 1500 were directly modeled. Larger proteins were split into domains as described above, and all pairs of domains were modeled if none had a combined length > 1500. AlphaFold network outputs the ‘logits’ of ‘distogram’, which are the probability distributions for the distances between the Cβ atoms of two residues to be in a serial of distance bins (64 bins of equal size, from 2 to 22 Å). Residue–residue contact probability was calculated as the sum of the probabilities for the first 32 distance bins, corresponding to distances between 2 and 12 Å. For a pair of proteins, the matrix (m) of contact probability is of the shape $\left(L_{1}+L_{2}\right)$ by $\left(L_{1}+L_{2}\right),$ where $L_{1}$ and $L_{2}$ are the length of the first and second proteins, respectively. To investigate the interprotein contacts, we extracted the submatrix $m^{\prime }=m\left[0:L_{1}\right]\left[L_{1}:L_{1}+L_{2}\right]$. The highest residue–residue contact probability in this matrix $m^{\prime }$ is used as the contact probability for a pair of proteins.

We also applied two complex prediction methods developed based on AlphaFold: AF2Complex [38] and FoldDock [39]. These two methods mainly differ in the scoring of the complex. AF2Complex's metric is an interface score that depends on the predicted aligned error of interface residue pairs and the interface size. FoldDock relies on the pDockQ score based on the predicted LDDT (pLDDT) scores of interface residues and the number of contacts in the predicted interface. In comparison, our in‐house program (AF‐contact) ranks the complex models by the maximum intermolecular contact probabilities. We evaluated the performance of the three programs by comparing their predicted PPIs with the highest confidence to available experimental structural complexes (described below).

### Analysis of somatic missense mutations in RCC PPI interfaces

We collected genome‐level somatic mutations of RCC from the COSMIC database [81] and a recent cohort study of RCCs [47]. We used BindProfX [48] with FoldX energy function [49] to predict the free energy change of mutations mapped to protein–protein interaction interfaces in experimentally determined protein complex structures and AlphaFold models with confident predictions (contact probability > 0.9). Experimental structural complexes were obtained from bioassemblies of entries in the RCSB PDB database. We considered all pairwise interactions with at least 10 inter‐residue contacts at the interface (a contact is defined as two residues from different chains within a distance of < 8 Å). Difference between the wild‐type and mutant in free energy changes (ΔΔG) above 1.4 kcal·mol^−1^ is considered to have a destabilizing effect on the binding affinity of the complex. The 1.4 kcal·mol^−1^ cutoff is more stringent than the absolute value of the threshold suggested as stabilizing mutations (−1 kcal·mol^−1^) by the BindProfX program [48] (https://zhanggroup.org/BindProfX/help.html). The free energy difference of 1.36 Kcal·mol^−1^ corresponds to about a 10‐fold change (1 order of magnitude) in binding affinity at room temperature. Other studies have used similar cutoff values ranging from 1 to 1.5 Kcal·mol^−1^ when applying the BindProfX program [89, 90]. Of the 996 RCC somatic mutations mapped to experimental structures or AlphaFold models, about 27% (268 mutations) have ΔΔG > 1.4 kcal·mol^−1^ (Table S2). Additional 88 mutations with ΔΔG in between 1 kcal·mol^−1^ and 1.4 kcal·mol^−1^ (Table S2) may also have destabilizing effect on the binding affinity of the complex.

## Conflict of interest

The authors declare no conflict of interest.

## Author contributions

JP, JZ, and QC contributed to conceptualization and methodology; JP contributed to formal analysis and investigation and writing—original draft preparation; QC contributed to writing—review and editing and supervision.

## Supporting information


**Table S1.** Top 1000 protein complexes predicted by AF‐contact, AF2Complex, and FoldDock.

**Table S2.** Differences of free energy changes of mutations in the interfaces of AlphaFold‐predicted protein complexes and experimentally determined protein complexes.

## Data Availability

The structural models of AlphaFold‐predicted complexes (in PDB format files and in PyMOL sessions) are available at: http://conglab.swmed.edu/RCC.
